# Mass Spectral Analyses of *Salmonella* Myovirus SPN3US Reveal Conserved and Divergent Themes in Proteolytic Maturation of Large Icosahedral Capsids

**DOI:** 10.3390/v15030723

**Published:** 2023-03-10

**Authors:** Aaron Scheuch, Sara A. M. Moran, Julia N. Faraone, Sophia R. Unwin, Gialinh Vu, Andrea Denisse Benítez, Nurul Humaira Mohd Redzuan, Dana Molleur, Sammy Pardo, Susan T. Weintraub, Julie A. Thomas

**Affiliations:** 1 Thomas H. Gosnell School of Life Sciences, Rochester Institute of Technology, Rochester, NY 14620, USA; 2 Department of Biochemistry and Structural Biology, The University of Texas Health Science Center at San Antonio, San Antonio, TX 78229, USAweintraub@uthscsa.edu (S.T.W.)

**Keywords:** myovirus, *Salmonella*, prohead protease, proteolytic maturation, mass spectrometry

## Abstract

*Salmonella* myovirus SPN3US has a T = 27 capsid composed of >50 different gene products, including many that are packaged along with the 240 kb genome and ejected into the host cell. Recently, we showed that an essential phage-encoded prohead protease gp245 is responsible for cleavage of proteins during SPN3US head assembly. This proteolytic maturation step induces major changes in precursor head particles, enabling them to expand and undergo genome packaging. To comprehensively define the composition of the mature SPN3US head and elucidate how it is modified by proteolysis during assembly, we conducted tandem mass spectrometry analysis of purified virions and tailless heads. Fourteen protease cleavage sites were identified in nine proteins, including eight sites not previously identified in head proteins in vivo. Among these was the maturation cleavage site of gp245 which was identical to the autocleavage site we had previously identified in purified recombinant gp245. Our findings underscore the value of employing multiple mass spectrometry-based experimental strategies as a way to enhance the detection of head protein cleavage sites in tailed phages. In addition, our results have identified a conserved set of head proteins in related giant phages that are similarly cleaved by their respective prohead proteases, suggesting that these proteins have important roles in governing the formation and function of large icosahedral capsids.

## 1. Introduction

The mature capsid, or head, of all tailed phages is a thin icosahedral shell formed by the major capsid protein whose role is to protect the phage genome [1]. At one vertex of the capsid is a dodecameric structure formed by the portal protein that has essential roles in head assembly, genome packaging and tail attachment [2,3,4]. Although there are conserved structural folds within the major capsid and portal proteins of all tailed phages, there is considerable diversity in the sequences of these proteins between different phages, as well as surprising variability in the dimensions and composition among the heads of different phages [5]. Recent studies have shown that phages with unusually long genomes >200 kb are prevalent in the environment [6]. To package and protect such long genomes, those phages have heads with high triangulation or T numbers [5], thus earning them the monikers “giant” [7,8] or “jumbo” [9] phages. Mass spectral studies of several representative giant phages have shown that their virions are surprisingly complex and can be formed from >70 different proteins—more than double the number of different proteins in many shorter genome phages whose capsids have correspondingly smaller T numbers [10,11,12]. Since many of the head proteins of giant phages are uncharacterized, there are numerous missing details regarding the assembly and function of their heads.

Our research on the *Salmonella* myovirus SPN3US has demonstrated that its 240 kb genome is packaged into a T = 27 head. This large structure consists of an outershell formed from 1615 copies of the major capsid protein (gp75), and many (>50) other proteins [13,14]. To give some context as to how unusual the heads of large phages such as SPN3US are relative to many other tailed phages, the model phages P22 (a podovirus) and HK97 (siphovirus) each have T = 7 heads formed from 415 copies of their respective major capsid proteins and virions with a total of nine different proteins [15,16]. What makes the heads of large phages even more unusual is that along with the DNA, there is also a large amount of protein within them (estimated to be >40 MDa in SPN3US), the exact composition and structure of which are undefined [13,17]. It is likely that many of the internal head proteins of SPN3US enter the *Salmonella* cell and have important roles during infection based on the precedents of head ejection proteins in other phages, such as T4, P22 and T7. In addition, among the SPN3US internal head proteins is an essential five-subunit RNA polymerase that logically must be ejected into the cell for the transcription of early phage genes [14]. Other potential roles for SPN3US’s internal head proteins include guiding head assembly or contributing to the stability of the mature head structure.

To better understand the composition of the SPN3US virion, we undertook a mass spectrometry analysis of the wild-type (WT) phage [17]. This initial study indicated that at least seven head proteins are cleaved by its prohead protease gp245 during head maturation [17], including the major capsid protein (gp75), the portal protein (gp81) and the highly abundant E proteins (gp53 and gp54). Evidence that gp245 undergoes auto-cleavage was obtained by mass spectrometry of purified and active recombinant gp245 [17]. All of the identified gp245 cleavages were determined to be *C*-terminal to the sequence motif AXE-, where X can be any amino acid. These findings suggested that proteolytic maturation is an important event during SPN3US head formation. The essential nature of this event was confirmed by the isolation and characterization of several protease mutants that displayed conditional-lethal phenotypes [18]. When the protease mutants were grown under non-permissive conditions, head formation stalled and precursor head particles (proheads) remained bound to the inner host membrane. Proteomic analyses of purified proheads from those mutants indicated that gp245 was not present in their proheads, although gp245 would be expected to be present in wild-type (WT) heads. In addition, the proteins that had been previously identified as being cleaved by gp245 in the WT phage were not found to be cleaved in the mutant proheads, as evidenced by the fact that the propeptide regions of those proteins were intact [17,18].

Together, these results confirmed the importance of gp245 during head assembly, and support the contention that cleavage by gp245 is needed for processes such as prohead release from the inner membrane, head expansion, and genome packaging. These findings are also consistent with the concept that major head assembly steps in SPN3US are anciently derived and share similarities with those steps in the model *E. coli* phage T4 [17]. These similarities exist despite there being substantial differences in the head size and structure of SPN3US compared to T4 and are the result of both phages having diverged counterparts to four proteins with integral roles in head morphogenesis: the major capsid protein, portal, terminase and prohead protease [17].

Our previous studies also revealed that proteolytic maturation in SPN3US shares features with head maturation in the related *Pseudomonas* phages, PhiKZ and 201ϕ2-1—in particular, similarity between the cleavage specificity of the SPN3US prohead protease and that of the homologous proteases in PhiKZ and 201ϕ2-1 [11,12,19]. However, a higher number of head proteins were found by mass spectrometry to have been cleaved in PhiKZ and 201ϕ2-1 by their respective prohead protease (19 proteins in each phage) than identified for SPN3US. This raised the possibility that not all the substrates of gp245 had been identified in SPN3US due to limited protein sequence coverage for many proteins in that study. An alternative explanation would be that there might be substantial differences in the numbers of head proteins that undergo proteolytic maturation between related giant phages.

To more fully characterize the SPN3US head proteome, in this study we conducted comprehensive mass spectral analyses and employed multiple experimental strategies in order to increase the likelihood of identification of cleavage sites in SPN3US head proteins. The resulting data showed that the SPN3US head is composed of 53 different proteins and identified 14 prohead protease cleavage sites in nine proteins, including eight sites not previously identified in head proteins in vivo. Additionally, we obtained convincing evidence that a large proportion (~80%) of the remaining SPN3US head proteins do not undergo cleavage by gp245. These findings demonstrate that considerable differences in head maturation events can exist among related phages. We also identified a core set of seven conserved proteins that undergo cleavage by the prohead protease in related phages which represent excellent foci for future research.

## 2. Materials and Methods

### 2.1. Isolation and Genome Sequencing of SPN3US Mutant am107

SPN3US mutant am107 was isolated by mutagenesis of the wild-type phage as described previously [14]. Genome sequencing of SPN3US mutant am107 was conducted on an Illumina MiSeq (150 bp paired-end reads) at the University of Rochester Genomics Research Center. Assemblies and mutation analyses were performed using SeqMan NGen and SeqMan Pro, respectively (DNAStar). GenBank accession No. JN641803.1 was used as the reference genome.

### 2.2. Preparation of Purified SPN3US Virions and Heads

Wild-type SPN3US virions were prepared from a single plaque that had been resuspended in SM buffer [100 mM NaCl, 8 mM MgSO_4_, and 50 mM Tris-Cl (pH 7.5)]. Briefly, ~10^5^ pfu of the phage suspension was plated with exponential stage *Salmonella enterica* Typhimirium strain TT9079 in a soft LB broth overlays containing 0.34% agar, 1 mM MgCl_2_ and 1 mM CaCl_2_ on LB bottom plates. After overnight incubation at 30 °C, the overlays were harvested and diluted approximately two-fold in SM buffer with lysozyme (~2 mg/mL final concentration) and left overnight at 4 °C. The phage mixture was clarified and concentrated by differential centrifugation (7500 rpm, 10 min and then 18,000 rpm, 30 min, at 4 °C in a Beckman JA25.50 rotor) and the phage pellet resuspended in SM buffer overnight at 4 °C.

SPN3US heads were prepared from a liquid infection of mutant am107 at an MOI of 10 in the *S.* Typhimurium strain TT9079 (a non-permissive host) in LB + N broth at 33–34 °C. At ~25–30 min post infection, cells were centrifuged (5000 rpm, room temperature), and resuspended in fresh media to remove any unadsorbed phage. After 2.5 h, lysozyme (2 mg/mL) was added to the infected-culture and incubated for 30 min at room temperature. The mixture was then subjected to differential centrifugation as described for the WT virions above, and the pellets containing the head particles resuspended overnight in SM buffer at 4 °C.

The resuspended WT virion and head samples were each subjected to cesium chloride (CsCl) step gradient and then CsCl buoyant density gradient purification. Samples (800 μL) were layered onto CsCl step gradients composed of the following concentrations of CsCl: 1.59 g/mL (1 mL), 1.52 g/mL (1 mL), 1.41 g/mL (0.9 mL), 1.30 g/mL (0.9 mL), and 1.21 g/mL (0.9 mL). The buffer used throughout the gradient contained 10 mM Tris-HCl (pH 7.5) and 1 mM MgCl_2_. The tubes were centrifuged at 108,000× *g* for 3 h at 4 °C in an LE-80K ultracentrifuge (Beckman Coulter Life Sciences, IN, USA), and the resulting bands were harvested by side tube puncture. The refractive index of each sample was measured using a refractometer, and then the sample was added to a freshly prepared solution of 10 mM Tris-HCl (pH 7.5) and 1 mM MgCl_2_ containing CsCl at the refractive index of that sample. The buoyant-density gradients were then subjected to centrifugation at 108,000× *g* for 18 h at 4 °C. Bands containing either WT virions or tailless heads were collected by side tube puncture, and dialyzed against three changes of 50 mM Tris-Cl (pH 7.5), 200 mM NaCl, and 10 mM MgCl_2_ at 4 °C to remove remaining cesium chloride.

### 2.3. Gel Electrophoresis and Mass Spectrometry Analysis of SPN3US Virions and Heads

**Figure 1:**
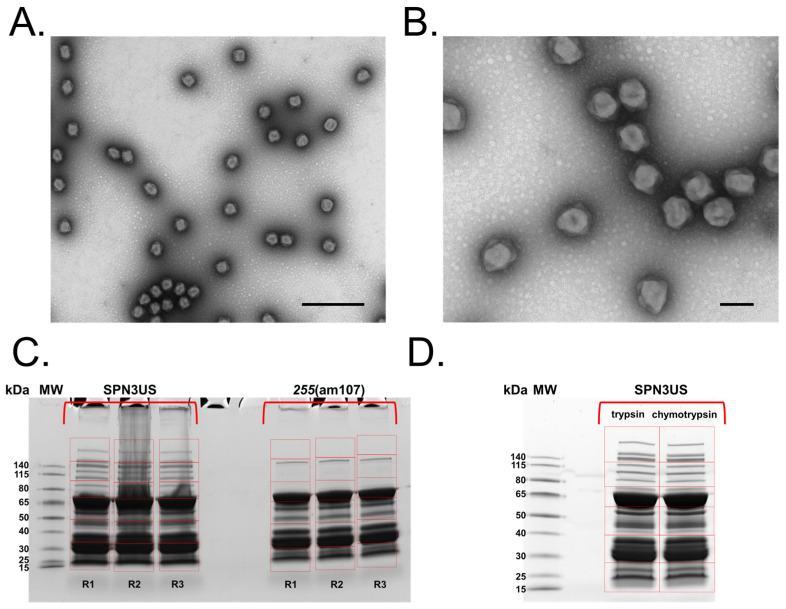
Transmission electron microscopy (TEM) and 1-D SDS-PAGE of *Salmonella* phage SPN3US. (**A**,**B**) TEM of head particles produced by *Salmonella* phage SPN3US mutant *255*(am107) grown under non-permissive conditions and stained with uranyl acetate. Space bars represent 500 nm (**A**) and 100 nm (**B**). (**C**,**D**) 1-D SDS-PAGE of SPN3US. Gel slices excised for mass spectrometry analysis are indicated in red. (**C**) 1-D SDS PAGE of replicate samples (R1-R3) of wild-type SPN3US and heads from mutant *255*(am107). Proteins were analyzed on a Thermo Fisher Orbitrap Fusion Lumos mass spectrometer after digestion with trypsin (**D**) Wild-type SPN3US proteins were digested with either trypsin or chymotrypsin prior to mass spectrometry analyses on a Thermo Fisher Orbitrap Fusion Lumos mass spectrometer.

**Figure 2:**
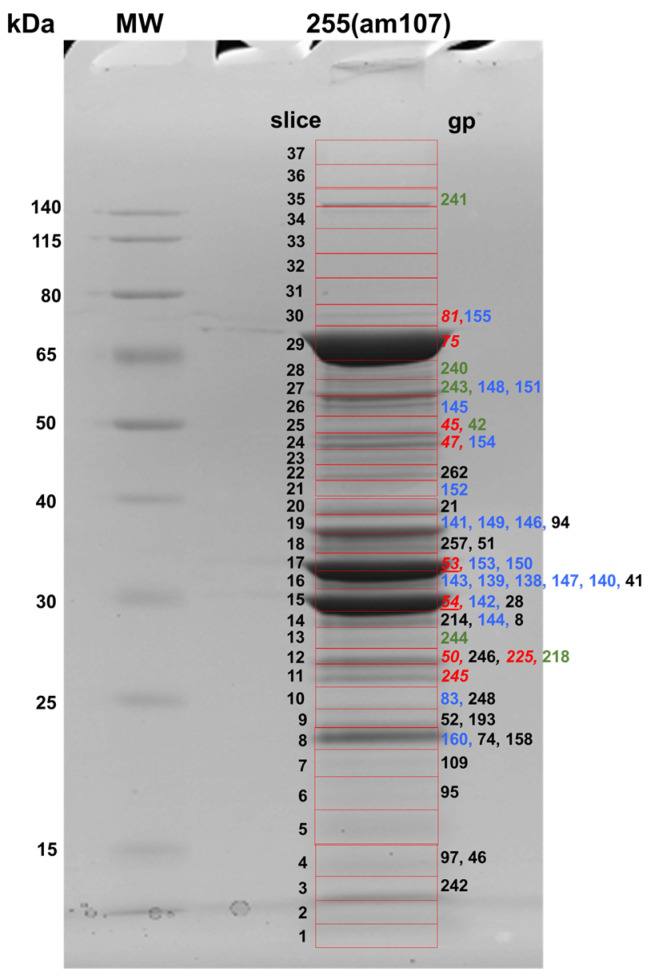
SDS-PAGE separation of proteins in purified head particles from SPN3US mutant *255*(am107). Gel slices excised for mass spectrometry analysis are indicated in red. Identified SPN3US proteins are indicated to the right of each slice where the maximum number of peptide spectrum matches (PSMs) was identified for that protein. Protein lists are in order (left to right) of highest to lowest numbers of PSMs identified in the slice. Proteins cleaved by the prohead protease gp245 are indicated in red and italicized. Paralog family A proteins (gp53 and gp54) are underlined, paralog family B proteins are indicated in blue and the virion RNAP subunits are indicated in green. The mass spectrometry analyses were performed on a Thermo Fisher Orbitrap Fusion Lumos mass spectrometer.

Samples containing purified SPN3US virions or heads were boiled for 10 min in SDS sample buffer prior to electrophoresis on Criterion XT MOPS 12% SDS-PAGE reducing gels (Bio-Rad, Hercules, CA, USA); proteins were visualized by staining with colloidal Coomassie blue. Two different strategies were used for SDS-PAGE. For the gels with images shown in Figure 1, proteins were electrophoresed for approximately 1.5 cm and each lane was excised into six slices (“GeLCMS”). A full-length separation was used for the gel shown in Figure 2, and 37 slices were excised, isolating visually distinct bands whenever possible. Relatively high concentrations of phage proteins were loaded onto the SDS-PAGE gels to enhance mass spectrometry sequence coverage and localization of sites of proteolysis of phage proteins. After de-staining, proteins in the gel slices were reduced with TCEP [tris(2-carboxyethyl)phosphine hydrochloride] and then alkylated with iodoacetamide in the dark before digestion with trypsin (Promega, Madison, WI, USA). HPLC-electrospray ionization-tandem mass spectrometry was conducted on either a Thermo Fisher Orbitrap Fusion Lumos mass spectrometer or a Thermo Fisher LTQ Orbitrap Velos Pro mass spectrometer (as specified in corresponding figure legends); each was fitted with a New Objective PicoView NanoESI source. For both instruments, on-line HPLC separation was accomplished with an RSLC NANO HPLC system (Thermo Scientific/Dionex, Waltham, MA, USA): column, PicoFrit™ (New Objective, Littleton, CO, USA; 75 μm i.d.) packed to 15 cm with C18 adsorbent (Vydac, Columbia, TN, USA; 218MS 5 μm, 300 Å); mobile phase A, 0.5% acetic acid (HAc)/0.005% trifluoroacetic acid (TFA) in water; mobile phase B, 90% acetonitrile/0.5% HAc/0.005% TFA/9.5% water; gradient 3 to 42% B in 30 or 60 min as indicated; flow rate, 0.4 μL/min. For analyses on the Orbitrap Velos Pro, precursor ions were acquired in the orbitrap mass analyzer from *m/z* 300–*m*/*z* 2000 in centroid mode at 60,000 resolution (*m*/*z* 400); data-dependent collision-induced dissociation (CID) mass spectra of the six most intense ions in each precursor scan were acquired at the same time in the linear trap (30% normalized collision energy). For analyses on the Lumos, precursor ions were acquired in the orbitrap from *m*/*z* 300–*m/z* 1500 in centroid mode at 120,000 resolution (*m*/*z* 200); data-dependent higher-energy collisional dissociation (HCD) mass spectra were acquired at the same time in the linear trap using the “top speed” option (30% normalized collision energy).

Mascot (v 2.8.1; Matrix Science; London, UK) was used to search the MS data files against locally generated protein databases for SPN3US (273 sequences; 80,176 residues), *Salmonella* TT9079 (4542 sequences; 1,397,889 residues) in addition to a database of common contaminants (e.g., trypsin and human keratin; 247 sequences; 128,130 residues). Cysteine carbamidomethylation was set as a fixed modification and methionine oxidation and deamidation of glutamine and asparagine were considered as variable modifications. For trypsin digests, the proteolytic enzyme was specified as “semi-trypsin” (i.e., requiring cleavage *C*-terminal to lysine or arginine on only one end of a peptide), with two missed cleavage allowed. When chymotrypsin was used for digestion, the cleavage specification in Mascot was “no enzyme,” thereby allowing cleavage after any amino acid. Mass tolerances for all searches were peptide, ±20 ppm; fragment ion, ±0.8 Da. Subset searching of the Mascot output by X! Tandem, determination of probabilities of peptide assignments and protein identifications, and cross correlation of the Mascot and X! Tandem results were accomplished by Scaffold 5 (v 5.2.1; Proteome Software, Portland, OR, USA). The Mascot output files were processed individually in Scaffold for the 37-gel slice experiment while for all other datasets, the files for an entire gel lane were combined via the “MudPIT” option in Scaffold. Quality filters used in Scaffold for viewing and exporting the proteomics results were peptide, 99.9%; minimum number of peptides, 2; protein, 99.9%. These settings resulted in a protein-level FDR of <1%.

## 3. Results

### 3.1. Isolation and Electron Microscopy of a Tail Tube Mutant of SPN3US

The SPN3US mutant am107 was isolated via screening for mutants with a conditional lethal phenotype; the isolate had a high titer (≥10^11^ pfu/mL) when propagated on a serine suppressor strain of *Salmonella* (TT6675) and a reversion frequency ~10^−7^ when plated on the non-permissive strain (TT9079). Alignment of the reads obtained from Illumina genome sequencing against the SPN3US reference genome had an average coverage of 777X and revealed the presence of a nonsense mutation at position 226689 within the gene SPN3US_0255 (Table S1), confirming that this gene and its product, gp255, are essential. Gp255 is a major tail protein that forms the inner tube of the SPN3US contractile sheath. Based on precedents in other phage genetic systems (e.g., T4) in which tails assemble independently from heads and are only attached to the head after DNA packaging is completed [1,20], the *255*(am107) mutant was expected to form heads and no tails when grown under non-permissive conditions. This expectation was confirmed by TEM of purified particles from *255*(am107) stained with uranyl acetate which showed the presence of DNA-full heads and no tails (Figure 1).

### 3.2. Proteomic Analyses of the SPN3US Virion and Head

Tandem mass spectrometry analysis (Thermo Fisher Orbitrap Fusion Lumos (Waltham, MA, USA)) of a sample of dual CsCl gradient purified SPN3US yielded identification of 102 phage proteins in the WT virions (99.9% protein probability, 99.9% peptide probability, two peptides minimum, resulting in an FDR of <1/223 proteins) (Table 1 and Table S2). For this experiment, three aliquots of the WT virion sample (replicates R1, R2, and R3) were electrophoresed for approximately 1 cm on an SDS-PAGE gel and each lane was divided into six slices for digestion and MS analysis (Figure 1, Table S2). The identified proteins ranged in calculated molecular weight from 10.5 kDa (gp242, a protein of unknown function) to 259.1 kDa (gp239, tail tape measure protein). There was a wide distribution of the total numbers of peptide spectrum matches (PSMs) assigned to each protein, with the greatest number consistently identified for the major capsid protein, gp75 (Table S2). This is not unexpected since gp75 is the protein with the highest copy number in the virion (1615 copies) based on structural evidence [13]. Using our stringent confidence cutoffs, several proteins were detected with low numbers of total PSM and were not found in all replicate samples. [Examples can be found in Table S2]. Some of the proteins with low numbers of assigned PSMs may not be true virion proteins and may have been inadvertently “stuck” to the exterior of the virion and/or were trapped within the head during assembly. We refer to the SPN3US proteins that are not a true part of the final virion structure as non-virion proteins. Non-virion proteins typically have other roles in infection, such as DNA replication and repair, gene expression or lysis of the *Salmonella* cell.

The delineation of the SPN3US proteins identified in the purified virion samples in low amounts into true virion proteins (e.g., a protein that is only present in one copy per virion) versus non-virion proteins was challenging because only ~10% of the identified SPN3US proteins had known functions. The criteria used in this study to designate an SPN3US protein as a virion component were its identification in all replicates, with an average of ≥10 total PSMs, which is ~1000-fold less than the total number of PSMs identified for the major capsid protein. The formulation of these criteria was also guided by the identification of a single protein with a known non-virion function in the samples (gp100, the thymidylate kinase), for which an average of three PSMs was identified in the WT phage (Table S2). The expectation is that this strategy to designate SPN3US proteins as virion components would enhance the likelihood that non-virion proteins are not incorrectly assigned as virion proteins. Based on the criteria listed above, 83 SPN3US proteins were assigned as components of the WT virion.

The total number and identities of SPN3US proteins identified as components of the virion in this study are in good agreement with those we reported previously for virions that had been purified via a single CsCl gradient ultracentrifugation (Table S2) [17]. Of note, there were more *Salmonella* proteins identified in the current study than in the previous analyses, as can be seen in Table 1. However, the host proteins in this study were present at an approximately three-fold lower relative level in the dual gradient purified samples based on total numbers of PSMs identified for host and phage proteins. The identification of a higher number of different *Salmonella* proteins in the current study is likely a consequence of the enhanced detection limits and substantially faster scan speed of the Lumos mass spectrometer compared to the Orbitrap Velos Pro that was used in the earlier analyses of the WT virion [17]. The mass spectrometry results reported here indicate that addition of a second, longer, CsCl gradient centrifugation improved the overall purity of the phage preparation. Our results in this report also support that a single CsCl gradient purification can provide a good assessment of the composition of a phage virion for phages that are sensitive to lengthy purification regimes.

In order to classify the SPN3US virion proteins into head versus tail components, three replicates of purified tailless particles from *255*(am107) were analyzed in the same experiment as the WT phage, described above. Fifty-three proteins were assigned as components of the head and 30 as belonging to the tail, based on the logic that all head proteins would be present in both WT virions and head samples, but conversely, there should be no, or very little, tail proteins in the head samples given the indisputable genetic and structural evidence (Figure 1). To categorize head versus tail proteins, the numbers of PSMs for each identified protein were separately summed across the three replicates of the WT and the head samples and then each sum was normalized relative to the total of PSMs per experimental group. The ratio of the normalized PSMs for each protein in head versus WT samples were then calculated. A clear head versus tail assignment was apparent for almost all proteins. These included the following: (1) 53 proteins were designated as head proteins based on the numbers of normalized PSMs for them in the *255*(am107) heads being the same or greater than the numbers for the WT phage (Table S2). Consistent with this assignment, among those proteins are known head proteins, including the major capsid protein (gp75), the major E proteins (gp53 and gp54), and the subunits of the virion RNA polymerase (vRNAP). (2) Ten proteins were assigned as tail proteins based on their identification in the WT virion but not in the tailless particles. These proteins include known tail proteins, such as the tail sheath, baseplate and tail fibers (Table S2). (3) An additional 19 proteins were also determined to be excellent candidates for tail components because they were detected in *255*(am107) at levels of normalized PSMs that were <10-fold lower than in the WT phage (Table S2). Supporting the designation of these proteins as tail proteins was that among them was the tail sheath protein gp256, the tail tape measure protein gp239 and the members of the paralog C family (gps 168, 169 and 170) which are likely tail fiber/baseplate proteins based on their diverged homology to a peripheral baseplate protein of PhiKZ (gp131) [14,21]. (4) One lower abundance protein, gp38, was more difficult to definitively assign to a virion location because it was identified in the WT virion sample at ~ 1.5-times the normalized PSMs as in the head sample, but it was confidently detected in all three head-only sample replicates. The lower relative level of gp38 in particles without tails implies that it is in some way associated with the tail. As such, we tentatively assigned gp38 as a tail protein, bringing the total number of SPN3US tail proteins to 30 (Table S2).

The assignments of SPN3US head and tail proteins correlate well with designations that we previously derived from analysis of the mutant *64_112*(am27) that had amber mutations in both a virion gene (ORF64) and a non-virion gene (ORF112), and produced a tailless phenotype when grown under non-permissive conditions [14]. However, it is important to note that the function of the previously reported mutated genes is not known, and the heads produced from that mutant were more unstable than the heads of *255*(am107) during TEM (as judged by the need to employ an alternate negative staining protocol to observe intact head structures) [14]. Since the heads that were analyzed in this study were stable and derived from a well characterized mutated gene, our results led us to confidently revise the head–tail designations of three proteins: gp41, which is now assigned as a head protein, and gps 237 and 259, which are now designated as tail proteins. It is likely that some of the low abundance virion proteins (either head or tail) are actually components of the neck or connector structure that joins the head to the tail. Additional studies are needed to determine their exact locations.

In view of the fact that there were no tails observed in the micrographs of *255*(am107), it was somewhat unexpected to identify peptides for the tail sheath and tube proteins in *255*(am107) samples. We speculate that the low numbers of PSMs identified for gp256 in the *255*(am107) sample result from a strong affinity between gp256 and components of the connector structure, which in a WT infection has an important role in head–tail joining. In addition, a small number of PSMs (10) was identified for the tail tube protein (gp255) in one replicate sample (R1) of *255*(am107). Evidence from the mass spectrometry analyses indicates that a truncated form of gp255 was expressed.

### 3.3. Identification of Prohead Protease Substrates and Cleavage Sites

Our previous demonstration that the SPN3US prohead protease gp245 is essential for virion formation emphasized the need to more clearly define its role in head maturation. In order to enhance our ability to comprehensively detect gp245 cleavage sites in SPN3US (cleavage *C*-terminal to a glutamate [17]), we used several different strategies for analysis of phage heads and virions. Mass spectrometry experiments used for this purpose included (1) triplicate analyses of WT phage and heads from *255*(am107) using 1 cm SDS-PAGE separation and excision of each gel lane into six slices (“GeLCMS”), followed by tryptic digestion in preparation for MS analysis (results described above); (2) separation of proteins from *255*(am107) heads on a full-length gel followed by excision of 37 slices and tryptic digestion (Figure 2, Table S3); and (3) GeLCMS (six slice) analyses as employed for the heads and tails in (1) above, starting with an independently purified sample of the WT phage in conjunction with parallel digestion with trypsin and chymotrypsin prior to mass spectrometry analysis (Figure 1, Table S4). Loading of relatively high levels of total protein for SDS-PAGE and analysis using high-performance mass spectrometers contributed to sufficient sequence coverage for confident detection of sites of prohead protease cleavage.

Specification of “semi-trypsin” (i.e., requiring cleavage *C*-terminal to a lysine or arginine on only one end of a peptide) for database searching of the MS files led to the identification of 14 prohead protease (gp245) cleavage sites in nine proteins (Table 2). Cleavage at each of the sites was consistent with the gel migration of the mature proteins identified in the 37-slice experiment. Among the cleavage sites were the six previously identified in the WT phage [17] (Table 2). Thirteen of the 14 cleavage sites result from removal of an *N*-terminal propeptide from eight head proteins. One site—the maturation cleavage site of the prohead protease gp245 where there is in vivo removal (via auto-cleavage) of a 64-residue *C*-terminal propeptide—was only identified when chymotrypsin was used as the proteolytic enzyme prior to MS analysis (Table 2). This site was first observed by mass spectrometry analysis of purified and active recombinant gp245, also using chymotrypsin digestion [17].

The results of the current study led to the discovery of a previously unrecognized substrate of gp245–gp225, a 22.3 kDa head protein of unknown function which has a 24-residue *N*-terminal propeptide. Another substrate of gp245, gp45, also has a relatively short *N*-terminal propeptide of 20 residues. All of the other *N*-terminal propeptides released by gp245 are considerably longer, ranging from 111 to 254 residues (Table 2). The longest of these is in the portal protein gp81 whose maturation cleavage site was also only detected when chymotrypsin digestion was employed. A long propeptide (137 residues) was also identified for the highly abundant E protein gp54. The location of the maturation cleavage in gp54 results in it having a slightly longer propeptide than gp53 (125 residues), explaining why the mature fragment of gp54 migrates at a lower apparent molecular weight on SDS-PAGE than gp53 (Figure 2). We had previously predicted gp54 to have a 124-residue propeptide based on a sequence alignment of gp54 with its paralog gp53 [17]. The results in this study showed that gp54 is also cleaved at that position (E-124), providing evidence that the gp54 propeptide is cleaved into smaller peptide fragments.

The sites for cleavage of SPN3US proteins by gp245 reported in this study were consistently detected across the various experiments (Table 2). However, as noted above, chymotrypsin needed to be used for proteolytic digestion for identification of two of the maturation cleavage sites—the sites on gp81 and gp245. In addition, use of chymotrypsin was necessary to reveal the AQE-124 cleavage in gp54 (see above) via a low-abundance gp245-cleaved peptide. Our results highlight challenges associated with identifying in vivo cleavage sites, especially for low copy number proteins, in that cleavage site-defining peptides are frequently detected at a lower frequency and with lower relative abundance than many of the other assigned peptides.

**Figure 3:**
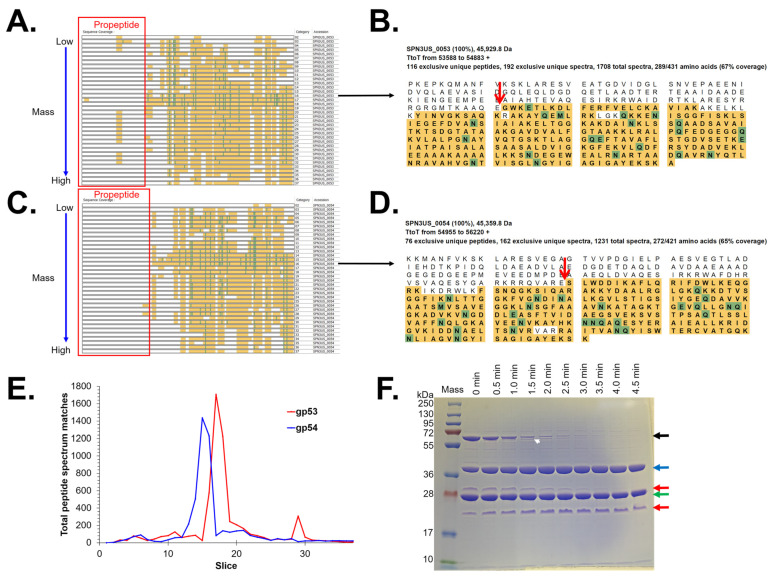
Proteolytic cleavage of SPN3US E proteins gp53 and gp54. (**A**) Sequence coverage map for gp53 in the 37-slice 1-D SDS-PAGE separation of head particle proteins (Figure 2). (**B**) Protein sequence coverage for gp53 in slice 17 showing its maturation cleavage site to generate the semi-tryptic peptide (E)GWKETLKDLFER(F) [red arrow; 57 peptide spectrum matches (PSMs) were assigned to that peptide]. (**C**) Sequence coverage map for gp54 in the 37-slice 1-D SDS-PAGE separation of head particle proteins (Figure 2). (**D**) Protein sequence coverage for gp54 in slice 16 showing its maturation cleavage site to generate the semi-tryptic peptide (E)SLWDDIK(A) [red arrow; 18 peptide spectrum matches (PSMs) were assigned to that peptide]. The maximum number of PSMs for gp54 is in slice 15—in agreement with the location shown in Figure 2. (**E**) Plot of the total PSMs assigned for gp53 and gp54 per gel slice. (**F**) Cleavage of gp53 by recombinant gp245 in vitro at room temperature. Black arrow, full-length gp53; red arrows, propeptide fragments of gp53, blue arrow, mature form of gp53; white arrow, a high molecular mass fragment of gp53 that may be the product of cleavage at ARE-12 (see Table 2).

**Figure 4:**
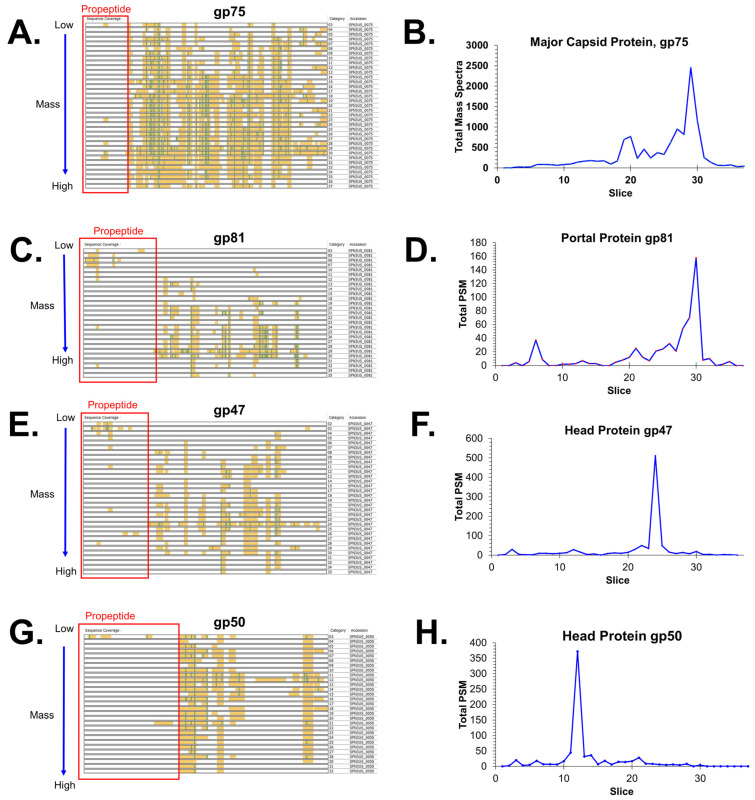
Mass spectrometry identification of SPN3US head proteins that are cleaved by the prohead protease (gp245) in *255*(am107). (**A**,**C**,**E**,**G**) Sequence coverage maps for the indicated proteins in the 37-slice 1-D SDS-PAGE separation of head particle proteins (Figure 2) (**A**) major capsid protein, (**C**) portal protein, (**E**) gp47, and (**G**) gp50. Plot of the total peptide spectrum matches (PSMs) assigned per gel slice for: (**B**) major capsid protein, (**D**) portal protein, (**F**) gp47 and (**H**) gp50.

Results of our mass spectrometry analyses indicated that for most of the processed head proteins, the propeptide regions were no longer detectable in the particles that were analyzed. This is not surprising as it is a general expectation in tailed phages that propeptides produced by a prohead protease exit the head during proteolytic maturation [22,23,24,25]. However, for several SPN3US head proteins, such as gp53 and gp81, higher numbers of PSMs were identified for peptides within their propeptide regions (e.g., Figure 3 and Figure 4), indicating that for those proteins, either some of the molecules were not processed and/or the resulting cleaved regions were not totally cleared from the capsid during maturation. These data were important for demonstrating that the removal of the long propeptides from SPN3US head proteins is a result of multiple cleavages of those propeptides, and not a single maturation cleavage. However, it is not known whether incomplete cleavage and/or removal of any propeptide in the SPN3US head impacts the infectivity (whether partially or fully) of the virion. This is because we do not have methodology to assess the proportion of heads that fall into those categories of having incomplete cleavages and/or incomplete removal of propeptides. Clearly, defining what causes there to be incomplete cleavage or incomplete removal of head protein propeptides, and subsequent impact on infectivity, are important questions to address in future studies and could have far-reaching implications (e.g., in the design of synthetic phages for therapy).

**Figure 5:**
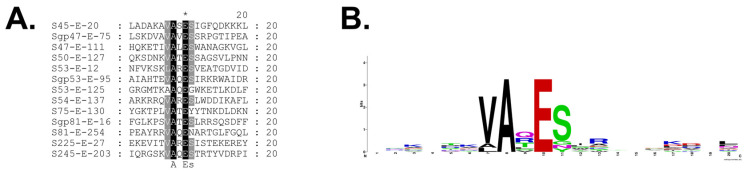
Cleavage specificity of the SPN3US prohead protease (gp245). (**A**) Alignment of the regions incorporating 10 residues upstream to 10 residues downstream of the cleavage site in SPN3US head proteins that are cleaved by gp245. Gp245 cleaves *C*-terminal to a glutamate residue (indicated with an asterick). (**B**) Sequence logo of the SPN3US cleavage sites generated by WebLogo (https://weblogo.berkeley.edu/logo.cgi, accessed on 4 January 2023).

Generation of a logo of the sequences flanking the gp245 cleavage sites based on our current data confirmed our earlier findings that gp245 cleaves *C*-terminal to the motif A-X-E (at positions P3-P2-P1), where X is any amino acid (Figure 5). In addition, there is a preference for valine at position P4 (and to a lesser degree alanine), and for a serine at position P1′. It is important to note that this logo may not be based on the sequences of all of the gp245 cleavage sites because there could be additional cleavage sites within the propeptide regions of some proteins that were not detected in this study. This possibility is supported by our observation of the presence of multiple sequence motifs that conform to the gp245 cleavage motif in the propeptides of several proteins, including in the gp53 propeptide. For gp53, we obtained mass spectral evidence to support that at least three of those motifs are cleaved; however, determination of whether there are additional sites cleaved in gp53 requires further experimentation. The observation of fragments of gp53 produced in vitro by the action of recombinant gp245 supports the validity of this type of in vitro experiment for future identification of additional cleavage sites in the cleaved SPN3US head proteins (Figure 3F). These types of in vitro experiments would also be suitable for the identification of factors that determine whether cleavage of propeptide regions goes to completion or not (see above). Additional SPN3US proteins may also be cleaved by gp245, as evidenced by the fact that there was protein sequence coverage of gp94 and gp262 that was *C*-terminal to residues that conform to the gp245 cleavage motif.

It was somewhat unexpected that our mass spectrometry results indicated that 42 SPN3US head proteins are not cleaved by gp245 (Table S6). This determination was based on several lines of evidence, including protein sequence coverage that precluded the possibility of cleavage; protein migration on 1-D SDS-PAGE at a position that was acceptably consistent with its predicted molecular weight and absence of sequences that conform to the gp245 cleavage motif. With regard to protein sequence coverage, we especially focused on protein *N*-termini, since, other than the propeptide produced by its self-cleavage, all the identified gp245 cleavages produce *N*-terminal propeptides. The *N*-termini of 24 proteins were identified (i.e., part of the mature protein) based on protein sequence coverage that initiated at the predicted *N*-terminal methionine (10 proteins) or at the second predicted amino acid residue but not the *N*-terminal methionine (14 proteins) (Table S6). The latter 14 proteins are likely to have been cleaved by the *Salmonella* methionine aminopeptidase (MAP) based on the amino acid occupying position P1′ in all those proteins being one of the amino acids required for the cleavage of cellular proteins by MAP (glycine, alanine, serine, threonine, cysteine, proline or valine) [26]. In addition, cleavage of the *N*-termini of 23 proteins by gp245 would not have been expected since they did not have a site in their sequence that conforms to the gp245 cleavage motif.

Our examination of the sequence coverage of all proteins led to the determination that gp139 is 10 amino acids longer than predicted. This conclusion was made possible because the SPN3US protein fasta files that were employed for the mass spectral searches included amino acids that are *N*-terminal to the predicted start methionine up to the next terminator codon (i.e., so-called terminator to terminator sequences). This evidence about the *N*-terminus of gp139 caused us to examine the nucleotides upstream of the gp139 gene (ORF_0139) to determine if an alternate start site was supported by the genetic data. We found credible evidence that expression of ORF_0139 could initiate upstream of the predicted start site at an alternate start codon (GTG). Twelve bases upstream of this start site is a sequence (AGGAGG) that conforms to a strong prokaryotic Shine–Delgarno sequence, which has been proposed to help compensate for weak start codons [27,28]. The co-ordinates for the revised ORF_0139 are 116,201–117,043 in GenBank Accession JN641803. We note that the revised start site for gp139 represents the second such revision of an SPN3US head protein based on mass spectral evidence, as we identified that the start site of gp47 gene was four codons upstream of its predicted site in our earlier analyses of the WT virion [17]. These findings underscore the value of mass spectrometry evidence to confirm and/or enhance the annotation of phage genomes.

## 4. Discussion

In this study, we undertook multiple analyses of SPN3US using high-performance mass spectrometry for comprehensive identification of the substrates of the prohead protease since it was not known from previous studies if all had been identified. Comparison of the proteomes of highly purified tailed versus tailless head particles revealed that the SPN3US virion is composed of 83 different proteins, 53 of which are part of the head and 30 of the tail. High levels of protein sequence coverage were obtained for many SPN3US head proteins in the nine mass spectral analyses which together identified 199,950 peptide spectrum matches (PSMs). The latter facilitated the identification of high confidence semi-tryptic peptides as robust evidence of cleavages of SPN3US head proteins by its prohead protease gp245. A total of 14 gp245 cleavage sites were identified in nine substrates, doubling the number of known cleavage sites. Eight substrates of gp245 have *N*-terminal propeptides, whereas gp245 itself has a *C*-terminal propeptide (Table 2). Two of the *N*-terminal propeptides are relatively short (20 and 24 residues in gp225 and gp45, respectively), the other propeptides are considerably longer (111–254 residues), underscoring the extent by which the SPN3US head is modified by proteolytic maturation. The longest propeptide is from the portal protein gp81, whose maturation cleavage site was one we had sought for an accurate calculation of its mature mass as this information is needed for future analyses to determine the copy number of SPN3US head proteins.

An unanticipated finding of our analyses of the SPN3US head proteome was the determination that a large proportion (~80%) of its proteins are not cleaved by the prohead protease. This result was unforeseen based on the related phages PhiKZ and 201phi2-1 each having at least 19 head proteins (~2-fold the number identified in SPN3US) that are cleaved by their respective proteases [11]. In addition, all three phages have homologous major head proteins, including the prohead protease, and other head proteins (Table 3). Therefore, our results demonstrate that considerable variability in the number of proteins that undergo proteolytic maturation can exist between related phages. The basis for that variability is unclear, but may be related to the activity of the prohead protease of each phage. Possibly, the SPN3US prohead protease has more stringent sequence requirements, as indicated by its cleavage logo being considerably less variable than those of the related phages.

**Figure 6:**
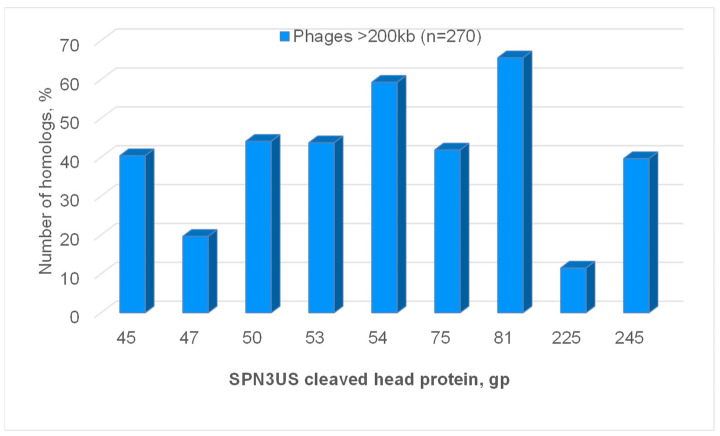
Numbers of homologs to cleaved SPN3US head proteins in long >200 kb genome phages identified by BlastP.

Intriguingly, the SPN3US head proteins that are cleaved appear to be conserved in numerous giant phages based on matches identified by BlastP (Figure 6). Homologs to the SPN3US portal and major capsid protein were found in 66 and 42%, respectively, of 270 phages with >200 kb genomes (Figure 6). This is not surprising as all tailed phages must have a portal and major capsid protein, although homologs between different phages may not always be detected due to the high level of genetic diversity that can exist between phages. It is more notable that homologs to the other six cleaved SPN3US head proteins were found in 11% to 59% of the long genome phages (Figure 6). This finding indicates the need for further studies to determine if there is any correlation between the taxonomic status of those long genome phages and the presence/absence of homologs to SPN3US’s cleaved head proteins. A preliminary examination of four phages known to be related to SPN3US suggests that such a relationship exists. Counterparts to all the SPN3US cleaved head protein were identified in the phages PhiEaH2 and CR5, that are closely enough related to SPN3US to have nucleotide similarity to it, as well as homologs to a large proportion of SPN3US’s head proteins (Table 3). In addition, counterparts to seven of SPN3US’s cleaved head proteins were identified in the phages 201phi2-1 and PhiKZ which are more diverged to SPN3US as evidenced by their having practically no detectable nucleotide similarity to SPN3US and homologs to ~42–47% of SPN3US’s head proteins (Table 3). The two cleaved SPN3US head proteins for whom no homolog was identified in PhiKZ or 201phi2-1, gp47 and gp225, also had the least number of homologs among the 270 > 200 kb genome phages (Figure 6).

The seven cleaved SPN3US head proteins that are conserved in related giant phages potentially represent a core set of giant phage head proteins that all undergo proteolytic maturation causing comparable structural changes in all their heads. This is because the homologs to those proteins in PhiKZ are all cleaved, and those in 201phi2-1 are either cleaved, or expected to be cleaved, by their respective proteases [11,12]. In addition, many of the propeptides of those homologs in PhiKZ and 201phi2-1 are of comparable length, such as the long propeptides of their portal and major capsid proteins which represent evidence of substantial remodeling of their outer shells during proteolytic maturation. However, further studies are required to elucidate the exact role of those long propeptides, as well as the propeptides of other proteins. Potentially, the propeptides of cleaved giant phage head proteins have roles analogous to the propeptides of the cleaved head proteins of T4, whose head undergoes a proteolytic maturation step with déjà vu-like similarities to that in SPN3US. Functions for the T4 propeptides include targeting of immature proteins into the prohead core and recruitment of chaperonins to assist with protein folding and assembly of heads with the correct dimensions and architecture [29,30].

## 5. Conclusions

To better understand the proteome of the SPN3US head and the impact of the prohead protease on its formation, in this study we analyzed highly purified virions and heads using high-performance mass spectrometry. These data showed the SPN3US virion to be composed of 83 different proteins, of which 53 belong to the head and 30 to the tail, although a small number of each category are likely components of the head–tail connector. Our findings highlight the advantages of using mass spectrometry to assess the purity of phage samples for host protein contamination. Our results also demonstrate the challenges inherent to the identification of post-translational modification events in phage proteins and how these challenges can be mitigated by employing multiple experimental approaches. A total of 14 prohead protease (gp245) cleavage sites were identified in nine SPN3US head proteins, confirming that proteolysis causes a major transformation of the head during its assembly. Seven cleavage sites were newly identified, including that of a new substrate, gp225. The maturation cleavage site of gp245 was also identified, confirming that the specificity of recombinant gp245 is the same as gp245 in vivo. Importantly, our results show that not only is the SPN3US prohead protease conserved in related giant phages, a core set of substrates of the prohead protease are also highly conserved. Our research will continue to focus on these proteins as they likely have important roles in governing the formation and function of the SPN3US head.

## Figures and Tables

**Table 1:** Total numbers of *Salmonella* and SPN3US proteins and mass spectra identified in dual gradient purified virions and heads.

		Virions—Single Gradient Purified ^1^	Virions—Double Gradient Purified ^2^			Heads—Double Gradient Purified ^2^		
			**R1**	**R2**	**R3**	**R1**	**R2**	**R3**
**Number of Proteins**	***Salmonella***	24	51	52	55	46	51	46
	**Phage**	86	98	98	100	78	80	77
**Number of PSM**	***Salmonella***	459	338	384	421	238	260	243
	**Phage**	8507	26,892	26,216	26,569	19,288	18,990	18,496
**Salmonella PSM/Phage PSM, %**		5.40	1.26	1.46	1.58	1.23	1.37	1.31

^1^ Data published previously [17] and included for reference purposes. ^2^ R1–R3 refer to replicates 1–3, respectively.

**Table 2:** Identification of SPN3US head proteins cleaved by the prohead protease gp245 in purified virions and head particles. The identification of a semi-tryptic peptide(s) that supports a gp245 cleavage site in each mass spectral analyses is indicated.

gp	Predicted Mass (kDa)	Mature Mass (kDa)	Propeptide Mass (kDa)	Cleavage Site (s) ^1^	WT 10-Slice ^2^	WT 6-Slice, Replicates ^3,4^	Heads 6-Slice, Replicates ^3,4^	Heads 37-Slice	WT 6-Slice	WT Chym 6-Slice	Protein Function/Comment
*Digestion enzyme:*					*Trypsin*					*Chymotrypsin*	
**45**	50.3	48.2	2.2	ASE-20	Y	Y(3)	Y(3)	Y	Y	Y	Head, unknown function
**47**	63.3	50.7	12.5	AVE-75 ALE-111 *	Y N	Y(2) Y(3#)	Y(1) Y(3#)	Y Y	Y Y	N N	Head protein, essential but unknown function
**50**	41.5	25.6	15.8	ATE-127	Y	Y(3)	Y(3)	Y	Y	Y	Head
**53**	45.2	31.5	13.8	ARE-12 * AQE-95 * AQE-125	N N Y	Y(3) Y(3) Y(3)	N Y(3) Y(3)	N Y Y	N Y Y	N Y N	High abundance E protein
**54**	45.1	30.3	14.8	AQE-124 * ARE-137 *	N N	N Y(3)	N Y(3)	N Y	N Y	Y# N	High abundance E protein
**75**	83.9	70.4	13.5	ATE-130	Y	Y(3)	Y(3)	Y	Y	Y	Major capsid protein
**81**	100.2	72.3	27.9	ATE-161 AQE-254 *	Y N	Y(3#) N	Y(3) N	N N	Y N	Y Y	Portal
**225**	25.1	22.3	2.8	ARE-24 *	N	Y (3)	Y (3)	Y	Y	Y	Head, low abundance
**245**	30.7	23.4	7.2	AQE-203	N	N	N	N	N	Y	Prohead protease

^1^ All sites were identified in this study, with newly identified sites indicated with an asterisk. ^2^ Data published previously [17] and included for reference. ^3^ The number of replicates in which a cleavage site was identified is indicated in parenthesis. ^4^ Cleavage sites identified via semi-tryptic peptides with probabilities <95% are indicated with a hash tag (the mass spectrum for each of those sites was manually inspected to ensure those sites were credible).

**Table 3:** Conservation of cleaved SPN3US head proteins in related phages.

		*Erwinia* Phage PhiEaH2 (JX316028)	*Cronobacter* phage CR5 (JX094500.1)	*Pseudomonas* Phage 201phi2-1 (EU197055)	*Pseudomonas* Phage PhiKZ (AF399011.1)
**Nucleotide**	**Percent identity (%)**	75	69	83	85
	**Query coverage (%)**	65	23	<0.1	<0.1
**SPN3US proteins**	**All proteins (264)**	220	160	77	72
	**Head proteins (55)**	49	48	26	23
	**Head proteins minus the 21 Paralog B proteins (34)**	31	32	18	19
	**Processed proteins (9)**	9	9	7	7

## Data Availability

The data presented in this study are available in the Supplementary Material (Tables S1–S6) and the Scaffold files are available from the corresponding author.

## Supplementary Materials

The following supporting information can be downloaded at: https://www.mdpi.com/article/10.3390/v15030723/s1, Table S1: Mutations identified from genome sequencing of SPN3US mutant am107; Table S2: Mass spectrometry identification of proteins in replicate samples of wild-type (WT) SPN3US phage and head particles (am107); Table S3: Identification of proteins by mass spectrometry in SPN3US heads from *255*(am107).; Table S4: Identification of proteins by mass spectrometry in wild-type SPN3US particles after digestion with the althernate enzyme chymotrypsin.; Table S5: Mass spectral sequence coverage of proteins identified in the wild-type (WT) SPN3US and head (am107) particles.; Table S6: Details of SPN3US head proteins.
